# The dynamics of resting fluctuations in the brain

**Published:** 2015-12-04

**Authors:** Gustavo Deco

**Affiliations:** 1 Center for Brain and Cognition, Universitat Pompeu Fabra / ICREA, Barcelona, Spain

## 

The grand average functional connectivity (FC) of a resting brain captures properly the well- structured spatial correlations between different brain areas. Whole-brain-models explicitly linking spontaneous local neuronal dynamics with the tractography based anatomical structure of the brain are able to explain the emergence of those spatial resting correlations. Nevertheless, resting activity is not only spatially structured but also shows a very stereotypical temporal structure which is characterized by rapid transitions switching between a few discrete FC states across time. In this talk, we introduce a powerful theoretical framework, which allows us to demonstrate that resting functional connectivity FC dynamics (FCD) constrains more strongly the dynamical working point of whole-brain models. Furthermore, using a very general neural mass model based on the normal form of a Hopf bifurcation we are able to demonstrate that the temporal dynamics of resting state fluctuations emerges at the edge of the transition between asynchronous to oscillatory behavior. Even more importantly, at that particular working point the global metastability of the whole brain is maximized. By optimizing the spectral characteristics of each local brain node, we discover the dynamical core of the brain, i.e., the set of nodes, which drives the rest of the whole brain by oscillations.

